# Association of Inter-arm Blood Pressure Difference with Asymptomatic Intracranial and Extracranial Arterial Stenosis in Hypertension Patients

**DOI:** 10.1038/srep29894

**Published:** 2016-07-14

**Authors:** Yan Wang, Jin Zhang, Yuesheng Qian, Xiaofeng Tang, Huawei Ling, Kemin Chen, Yan Li, Pingjin Gao, Dingliang Zhu

**Affiliations:** 1 Research Center for Hypertension Management and Prevention in Community, Shanghai Key Laboratory of Hypertension, Shanghai Institute of Hypertension, State Key Laboratory of Medical Genomics, Ruijin Hospital, Shanghai Jiaotong University School of Medicine, Shanghai, China; 2 Department of Radiology, Ruijin Hospital, Shanghai Jiaotong University School of Medicine, Shanghai, China

## Abstract

Inter-arm blood pressure (BP) difference has been associated with ischemic stroke. Local atherosclerosis of stroke differ among vulnerable individuals, whereas intracranial arterial stenosis (ICAS) is more frequently affected Asians, and extracranial arterial stenosis (ECAS) is more prevalent among whites. We hereby sought to explore the association of inter-arm BP difference with ICAS and ECAS in stroke-free hypertensive patients in Chinese population. All the 885 subjects were evaluated of ICAS and ECAS through computerized tomographic angiography. Both arm BP was measured simultaneously by Vascular Profiler-1000 device. In the continuous study, ICAS was significantly associated with age, male, average brachial SBP, diabetes, anti-hypertensive treatment and inter-arm DBP difference. ECAS was associated with age, inter-arm SBP and LDL. In the categorical study, subjects with the top quartile of inter-arm DBP difference (≥4 mmHg) showed significantly higher risk of ICAS (OR = 2.109; 95% CI, 1.24–3.587). And the participants with the top quartile of inter-arm SBP difference (≥6 mmHg) showed significantly higher risk of ECAS (OR = 2.288; 95% CI, 1.309–3.998). In conclusion, we reported a diverse association of inter-arm SBP/DBP difference with the ICAS/ECAS. Inter-arm DBP difference might be the early symbol of ICAS in Chinese population, which need further verification in long-term cohort study.

Stroke is the second leading cause of death and the most common cause of adult disability worldwide^1^, with ischemic stroke as the predominant subtype in Chinese population. Atherosclerosis has been related to stroke for a long time, but the local expression and severity of atherosclerosis differ among vulnerable individuals, whereas intracranial arterial stenosis (ICAS) is more frequently affected Asians, blacks and Hispanics, extracranial arterial stenosis (ECAS) is more prevalent among whites^2^,^3^. The origin of the difference may be multiple, including genetic, environmental factors, and the different underlying disorders^4^. Hypertension is a worldwide health problem, which is especially common in China^5^, contributing to a high incidence of and mortality from stroke^6^,^7^, as well as for incidence of ICAS^8^. Therefore, high blood pressure (BP) may account for part of the distinctiveness of ICAS and ECAS in Chinese population.

A difference in BP readings between arms can be observed in various populations^9^, which is more prevalent in hypertension patients^10^. A great deal of evidence has indicated that inter-arm BP difference, especially SBP difference, is an independent risk factor of ischemic stroke^11^, left ventricular hypertrophy^12^, and other fatal and nonfatal cardiovascular events^13^. However, there is little study focusing on the relationship of inter-arm BP difference and asymptomatic ICAS or ECAS. Therefore, in the present study, we aim to investigate the association of both inter-arm SBP and DBP difference with ICAS and ECAS in stroke-free hypertensive patients in Chinese population.

## Method

### Study design

This study was undertaken within the framework of an ongoing cross-section and prospective study in China, which was a computerized tomographic angiography (CTA) based study of intra- and extracranial asymptomatic artery stenosis and stroke outcome in stroke-free hypertension patients. Participants of current study were recruited from hypertension outpatients who were identified in Xinzhuang Community hospital between May 2012 and December 2015, and then referred to Ruijin Hospital, a general hospital in Shanghai. Patients with hypertension were invited to participate in the study if they had ≥2 cardiovascular risk factors defined according to the Chinese hypertension guidelines^14^ and were willing to undergo an examination of brain using computerized tomographic angiography (CTA) and participate in long-term follow-up of their health. Hypertension was defined as blood pressure (BP) ≥140/90 mmHg, or taking antihypertensive medication. Those who had stroke, transient ischemic attack or atrial fibrillation identified from medical history was excluded. Those who were unfit for CTA examination because of iodine allergy were also excluded.

### Ethics Statement

The study protocol was approved by the ethics committee of Ruijin Hospital and written informed consent was obtained from all participants. All experiments were performed in accordance with relevant guidelines and regulations of ethics committee of Ruijin Hospital.

### Demographic and clinical measurements

Both arm BP was measured simultaneously by the use of the Vascular Profiler-1000 device (Omron, Kyoto, Japan), an oscillometric cuff technique. Trained technicians placed the pressure cuffs on both arms and performed the measurement after the subject had rested for ≈10 minutes in the supine position. The device simultaneously and automatically measures the both arm BPs twice, and then discards the first measurement and only stores the second one in the database.

Body weight and height were recorded with participants wearing light indoor clothing and no shoes. Clinical information was collected by interview, including smoking and drinking habits, current drug intake, personal history of diabetes, etc. Current smokers were defined as those who had smoked cigarettes on one or more days in the past 30 days. serum concentrations of total cholesterol (TC) and low-density lipoprotein cholesterol (LDL), were performed in the Central Laboratory of Ruijin Hospital (Shanghai, China) using the standard protocols.

### CTA protocol

CTA was performed with a 64-section helical CT scanner (GE FX/I, General Electric, Fairfield, CT) as previous described^15^. CTA acquisitions were obtained after a single bolus intravenous injection of 70 ml OptirayIoversol 320 into the antecubital vein at a rate of 3 ml/sec. Scanning covered the whole brain down to the level of aortic arch with 5-mm slice thickness. Images were reformatted in axial, sagittal, and coronal planes with 1.25-mm slice thickness. All images were read at a workstation with the software of AW4.4 vessel analysis independently by two experienced radiologist who were blinded to clinical data of the patients. Stenosis was defined as a lesion that decreased arterial internal diameter. The percentage of stenosis was calculated as the ratio of the diameter of the diseased artery at its most severe site divided by the diameter of a nearby normal segment. The intracranial arteries included intracranial segment of internal carotid artery and vertebral artery, basilar artery, anterior cerebral artery, middle cerebral artery and posterior cerebral artery. The extracranial arteries included extracranial segment of internal carotid artery and vertebral artery, external carotid artery, common carotid artery and subclavian artery. The greatest stenosis at an artery was chosen as being representative for each subject. The number of arteries with stenosis for each patient was also counted. The two radiologists had good agreement in the designation of stenosis (κ = 0.93, P < 0.001). All disagreements were reviewed and adjudicated by a senior radiologist to reach a consensus.

### Statistic Analysis

For database management and statistical analysis, we used SPSS software (version 13.0; SPSS Inc., Chicago, Illinois, USA). Descriptive statistics for patients with or without ICAS were compared using a Pearson Chi-square test for categorical variables and Student t test for continuous variables. In continuous analyses, stepwise logistic regression in forward conditional method was performed to test the association of inter-arm SBP/DBP (1 mmHg) difference and other risk factors with ICAS/ECAS. The variables tested in the equation included age, sex, body mass index, current smoking and drinking status, diabetes, low-density lipoprotein, average brachial SBP, heart rate, anti-hypertensive treatment, statin use, inter-arm SBP/DBP difference. In categorical analyses, we tested the association of quartiles of inter-arm SBP/DBP difference (≥6 mm Hg or ≥4 mm Hg) and increased inter-arm SBP and DBP differences (≥10 mm Hg or ≥5 mm Hg) with ICAS/ECAS by the adjustment for age, sex, body mass index, current smoking and drinking status, diabetes, low-density lipoprotein, average brachial SBP, heart rate, anti-hypertensive treatment, statin use. All *P* values were 2-tailed, and a *P* value of <0.05 was considered statistically significant.

## Results

### Characteristics of the Study Participants

Of the 885 participants, 418 had no ICAS or ECAS, 191 had ICAS only, 129 had ECAS only, and 147 had concurrent extraintracranial artery stenosis (Table 1). Comparing with the stenosis absent group, the patients with ICAS were older and with higher frequency of male, higher both arm SBP, higher inter-arm DBP difference, and higher frequency of diabetes and anti-hypertensive treatment. The patients with ECAS were older and with higher frequency of male, higher inter-arm SBP difference and higher LDL.

### Continuous Analyses

In order to detected all the independent risk factors associated with ICAS/ECAS, we performed a stepwise logistic regression in forward conditional method, including the variables of age, sex, body mass index, current smoking and drinking status, diabetes, low-density lipoprotein, average brachial SBP, heart rate, anti-hypertensive treatment, statin use, and inter-arm SBP/DBP difference (1 mmHg). Variables potentially associated with ICAS/ECAS were shown in Table 2. ICAS was significantly associated with age, male, average brachial SBP, diabetes, anti-hypertensive treatment and inter-arm DBP difference. While ECAS was significantly associated with age, inter- arm SBP difference and LDL.

### Categorical Analyses

The categorical analyses of inter-arm SBP/DBP and ICAS/ECAS were in Table 3. After adjustment for the abovementioned covariates, the risk of ICAS was independently associated with the inter-arm DBP difference (P = 0.048). Comparing with the lowest quartile, the subjects with the top quartile of inter-arm DBP difference (≥4 mmHg) showed significantly higher risk of ICAS (P = 0.006) with the corresponding OR = 2.109 (95% CI, 1.24–3.587). The risk of ICAS in patients with inter-arm DBP difference ≥5 mmHg was 92% higher (P = 0.014). On the contrary, there was no significant association was detected between ECAS and inter-arm DBP difference. But the participants with the top quartile of inter-arm SBP difference (≥6 mmHg) showed significantly higher risk of ECAS (P =  0.004) with the corresponding OR = 2.288 (95% CI, 1.309–3.998). The risk of ECAS in patients with inter-arm SBP difference ≥10 mmHg was 254% higher. Neither inter-arm SBP difference nor DBP difference was associated with presence of concurrent extraintracranial artery stenosis.

### Inter-arm BP and number/severity of ICAS/ECAS

**Figure 1:**
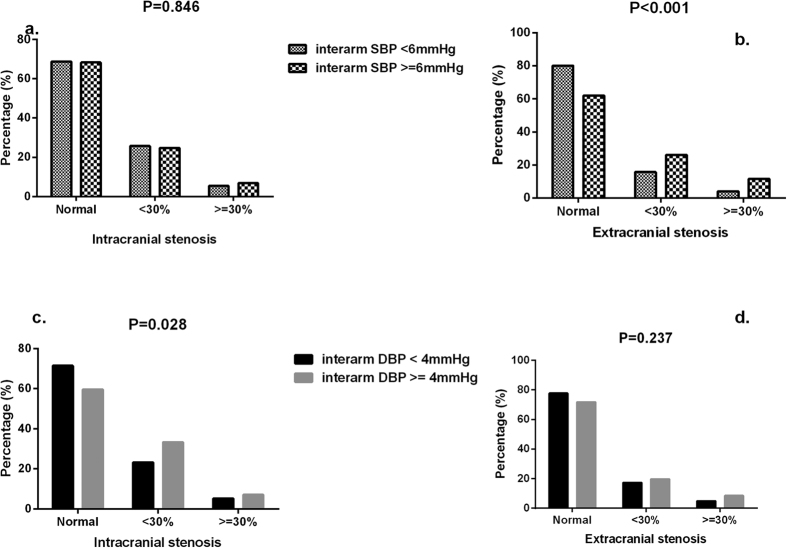
Prevalence of severity (%) of ECAS and ICAS stenosis according to top quartile of inter-arm SBP (**a**,**b**) and DBP (**c**,**d**). ECAS, extracranial arterial stenosis; ICAS, intracranial arterial stenosis.

Then, the severity of ICAS/ECAS was compared between patients with the top quartile of inter-arm SBP/DBP and the others. Comparing with the subjects with the inter-arm DBP <4 mmHg (5.3%), the subjects in the top quartile of inter-arm DBP difference (≥4 mmHg) had more moderate to severe ICAS (7.1%) (P = 0.028) (Fig. 1c). In contrast, comparing with the subjects with the inter-arm SBP difference <6 mmHg (4.1%), the subjects in the top quartile of inter-arm SBP difference (≥6 mmHg) had more moderate to severe ECAS (11.7%) (P < 0.001) (Fig. 1b).

**Figure 2:**
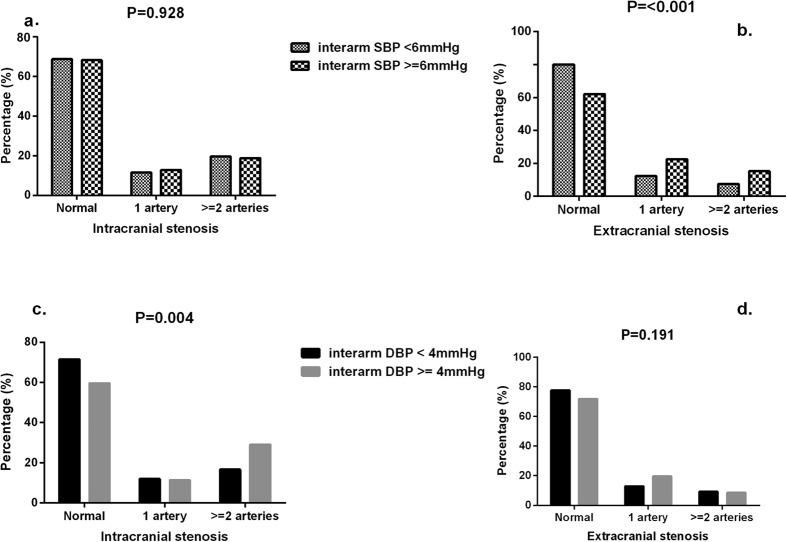
Prevalence of involved ECAS and ICAS arteries according to top quartile of inter-arm SBP (**a**,**b**) and DBP (**c,d**). ECAS, extracranial arterial stenosis; ICAS, intracranial arterial stenosis.

Moreover, patients in the highest quartile of inter-arm DBP difference showed higher frequency of multiple ICAS (29.1%) than the rest of participants (16.6%) (P = 0.004) (Fig. 2c). The patients in the highest quartile of inter-arm SBP difference showed higher frequency of multiple ECAS (15.3%) than the rest of participants (7.6%) (P < 0.001) (Fig. 2b).

Furthermore, the patients with inter-arm SBP difference ≥10 mmHg suffered more severe ECAS and involved ECAS vessels. While the patients with inter-arm DBP difference ≥5 mmHg suffered more severe ICAS and involved ICAS vessels (Supplementary Figs 1 and 2).

## Discussion

In this stroke-free Chinese hypertension population who underwent both intra- and extracranial CTA, a diverse association of inter-arm SBP/DBP difference with the ICAS/ECAS was detected. The inter-arm SBP difference was only significantly associated with the presence of ECAS and the severity of ECAS. On the contrary, the inter-arm DBP difference was only associated with the presence of ICAS and the severity of ICAS.

It was a very interesting thing that the different component of BP might relate to the stenosis in different part of the arterial tree separately. Although both ICAS and ECAS were process of atherogenesis and had the similar vascular risk factors, the distribution of atherogenesis varied among different populations, whereas patients of Asian possessed more ICAS^16^, while whites more frequently suffered from extracranial carotid lesions^17^. The genetic background heterogeneity might influence the atherogenesis at the different location of arteries. Uehara *et al*. had explored the risk factors of ICAS and ECAS in 425 stroke-free Japanese patients, they found that the independent predictors of ECAS were age, hyperlipidemia and ischemic heart disease (IHD), while those for ICAS were age, hypertension, diabetes mellitus and IHD^18^. Such results were in accordance with our findings, whereas we found that ECAS was associated with age and the concentration of LDL, and ICAS was related to age, sex, SBP, diabetes and the use of anti-hypertensive treatment. Although simultaneous hypercholesterolemia and hypertension management was suggested by several guidelines^19^, comparing with the ECAS which was more likely to be found by carotid ultrasonography and therefore drew more attention for anti-hypertensive and statin treatment, the patients with ICAS in the present study suffered higher BP and lower frequency of lipid lowering therapy. The uncontrolled BP and consequent augmented inter-arm DBP difference might cause the site specific atherogenesis, which was probably induced by various shear stress dependent endothelial gene expressions^20^, and diverse shear stress dependant accumulation of inflammatory cells in specific vascular regions^21^.

BP difference between arms was a common phenomenon which could be observed in various populations^22^, but the underlying mechanism of such difference between arms was still unclear^23^. There was an assumption that the asymmetrical arms arterial pressure was due to the atherosclerotic stenotic lesion^24^. And the relationship between inter-arm SBP and subclavian stenosis and cardiovascular mortality had been reported by a great deal of studies^25^. Exaggerated absolute SBP difference >10 mmHg or >15 mmHg has been associated with peripheral vascular disease, pre-existing cerebrovascular disease. In accordance with the former studies, we confirmed the association of inter-arm SBP difference with the ECAS, including the subclavian stenosis. Although the difference of DBP in two arms was also universal, there were limited studies analyzing the relation between inter-arm DBP differences and cardiocerebrovascular disorders. A study performed in general population found that exaggerated absolute inter-arm DBP (≥5 mmHg) but not inter-arm SBP was associated with left ventricular mass index^26^, Johansson *et al*. explained that the DBP might play more important role in the early phase of cardiovascular disease. In addition, Hu *et al*. had also found that inter-arm DBP difference but not SBP difference was associated with the flow-mediated dilatation of arm, which was an early index of arterial endothelium lesion^27^. The ICAS is the early lesion of cerebral vessels, and most of subjects in the present study suffered mild ICAS, so that the difference of inter-arm DBP might respond for early ICAS through disturbing the blood flow and subsequently influence the atherogenesis.

ECAS was the main lesion in the arterial tree in whites and was considered as an important risk factor for cardiovascular diseases. Inter-arm SBP difference had long been associated with ECAS, so that it was undoubtedly that the increased SBP difference between arms might be an indicator for fatal and nonfatal events in European population. However, ICAS accounted for 30–50% of ischemic stroke and >50% of transient ischemic attack in Asians^28^. Leng *et al*. had found that ICAS was not independently correlated with carotid intima-media thickness and conferred that atherosclerosis might start earlier in ICAS than in ECAS in Chinese population^29^. Therefore, Inter-arm DBP difference, as a possible indicator for ICAS in Chinese and other Asian population, should be paid more attention and deserved further researches.

We acknowledge that there were several limitations of the present study. First, as a cross-sectional study, we could not infer any causal relationship between inter-arm BP and ICAS/ECAS. Second, the majority of subjects were under antihypertensive treatment, which induced that the BP differences between arms were lower than the previous findings in hypertension population. But the prevalence of BP difference was similar with the results in general population^30^, and we had detected the association of inter-arm BP with ICAS/ECAS in both binary and continuous analysis. Third, nearly half of the participants had ICAS or ECAS, which seemed higher than other reports. The hypertension patients enrolled had at least two other risk factor, which might have higher prevalence of ICAS and ECAS than the common population. Moreover, most the involved vessels were with mild stenosis (<30%), and the prevalence of moderate to severe stenosis of intracranial arteries (15.2%) was similar to previous survey in high risk patients^31^. Forth, the ICAS was more common in Asians than the whites, so that our finding might not be directly generalized to other populations.

In conclusion, we found a diverse association of inter-arm SBP/DBP difference with the ICAS/ECAS. Inter-arm DBP difference might be the early symbol of ICAS in Chinese population. In addition, the further long-term cohort study was warranted to elucidate the concrete effect of inter-arm BP difference on the asymptomatic ICAS/ECAS.

## Additional Information

**How to cite this article**: Wang, Y. *et al*. Association of Inter-arm Blood Pressure Difference with Asymptomatic Intracranial and Extracranial Arterial Stenosis in Hypertension Patients. *Sci. Rep.* **6**, 29894; doi: 10.1038/srep29894 (2016).

## Supplementary Material

Supplementary Information

## Figures and Tables

**Table 1:** Clinical characteristics of hypertensive patients according to location of arterial stenosis.

	ICAS/ ECAS absent	ICAS present	ECAS present	COMB present	P
N	418	191	129	147	
Age (years)	62.9 ± 5.7	65.5 ± 5.9^†^	65 ± 5.6^†^	67.1 ± 5^†^	***<0.001***
Male (N,%)	150 (35.9)	101 (52.9)^†^	60 (46.5)*	90 (61.2)^†^	***<0.001***
Smoking (N,%)	52 (12.4)	34 (17.8)	21 (16.3)	26 (17.7)	0.224
Dringking (N,%)	57 (13.6)	34 (17.8)	15 (11.6)	21 (14.3)	0.420
Body mass index (kg/m^2^)	25 ± 3.1	25.3 ± 2.9	25 ± 3.2	25.4 ± 2.9	0.403
Left arm SBP (mmHg)	133.9 ± 15.4	138.8 ± 15.6^†^	134 ± 15.6	140 ± 16.9^†^	***<0.001***
Left arm DBP (mmHg)	79.1 ± 9.7	79.5 ± 9.3	77.3 ± 10.2	78.7 ± 8.9	0.218
Right arm SBP (mmHg)	134.9 ± 15.5	140.1 ± 15.6^†^	135.8 ± 16.1	141.3 ± 16.9^†^	***<0.001***
Right arm DBP (mmHg)	79.3 ± 9.5	79.7 ± 9.2	77.9 ± 10.3	79.2 ± 8.8	0.355
Inter arm SBP difference (mmHg)	3.2 ± 2.6	3.2 ± 2.6	4.2 ± 4.1^†^	3.7 ± 3.4*	***0.002***
Inter arm DBP difference (mmHg)	2.5 ± 2	3 ± 2.3^†^	2.8 ± 2.3	2.8 ± 2.4	***0.021***
Heart rate (beats/min)	71.9 ± 11.4	69.9 ± 10.5*	71.8 ± 11.7	71.6 ± 11.4	0.219
Plasma glucose (mmol/L)	5.1 ± 1.1	5.3 ± 1.6	5.2 ± 1.1	5.4 ± 1.4*	0.056
Total cholesterol (mmol/L)	4.9 ± 0.8	4.8 ± 0.9	5 ± 0.9	4.9 ± 0.9	0.154
Low-density lipoprotein (mmol/L)	2.8 ± 0.8	2.9 ± 0.8	3 ± 0.7*	3.1 ± 0.8^†^	***0.016***
Diabetes mellitus (N,%)	74 (17.7)	48 (25.1)*	24 (18.6)	39 (26.5)*	***0.047***
Anti-hypertensive treatment (N,%)	354 (84.7)	176 (92.1)*	117 (90.7)	135 (91.8)*	***0.014***
Statin use (N,%)	27 (6.4)	12 (6.3)	10 (7.8)	19 (12.9)*	0.068

Data are expressed as mean ± SD, or percentage (%). ECAS, extracranial arterial stenosis; ICAS, intracranial arterial stenosis; COMB, combined extra- and intracranial arterial stenosis. SBP, systolic blood pressure; DBP, diastolic blood pressure. P indicates the comparison among four groups. *p < 0.05 and ^†^p < 0.01 in comparison with ICAS/ ECAS absent group.

**Table 2:** The risk factors of intracranial and extracranial arterial stenosis.

	OR	95% CI	P
Isolated ICAS			
Age	1.063	1.028–1.098	<0.001
Male	1.872	1.296–2.704	0.001
Average brachial SBP	1.002	1.001–1.003	0.005
Inter-arm DBP difference	1.012	1.004–1.021	0.004
Diabetes	1.511	1.001–2.300	0.049
Anti-hypertensive treatment	0.539	0.291–0.998	0.048
Isolated ECAS			
Age	1.064	1.026–1.104	0.001
Inter-arm SBP difference	1.01	1.004–1.017	0.001
Low-density lipoprotein	1.417	1.078–1.862	0.012
COMB			
Age	1.129	1.083–1.178	<0.001
Male	2.757	1.780–4.270	<0.001
Average brachial SBP	1.002	1.001–1.003	0.003
Low-density lipoprotein	1.756	1.316–2.343	<0.001
Diabetes	1.712	1.042–2.812	0.034
Statin use	0.314	0.154–0.641	0.001

Stepwise logistic regression in forward conditional method was performed to test the risk factors of stenosis. The variables tested in the equation included age, sex, body mass index, current smoking and drinking status, diabetes, logw-density lipoprotein, average brachial SBP, heart rate, anti-hypertensive treatment, statin use, inter-arm SBP difference and inter-arm DBP difference. The odds ratio expressed the risk in the ICAS and ECAS group compared with the non-stenosis group. Isolated ECAS, extracranial arterial stenosis only; Isolated ICAS, intracranial arterial stenosis only; COMB, combined extra- and intracranial arterial stenosis; OR, odds ratio; SBP, systolic blood pressure; DBP, diastolic blood pressure.

**Table 3:** Associations of inter arm blood pressure difference with intracranial and extracranial arterial stenosis OR, odds ratio.

	Isolated ICAS			Isolated ECAS			COMB		
	OR	95% CI	P	OR	95% CI	P	OR	95% CI	P
Inter arm SBP quartiles			0.900			***0.003***			0.435
Q1 (<1 mmHg)	1	1–1		1	1–1		1	1−1	
Q2 (1–3 mmHg)	1.166	0.734–1.852	0.515	0.871	0.501–1.516	0.625	1.446	0.823–2.541	0.2
Q3 (3–6 mmHg)	1.034	0.609–1.758	0.900	0.938	0.496–1.772	0.843	1.517	0.819–2.809	0.185
Q4 (≥6 mmHg)	0.976	0.557–1.713	0.933	2.288	1.309–3.998	***0.004***	1.584	0.827–3.033	0.165
Inter arm SBP> = 10 mmHg	0.585	0.171–2.002	0.393	3.544	1.482–8.48	***0.004***	1.12	0.366–3.425	0.842
Inter arm DBP quartiles			***0.048***			0.495			0.128
Q1 (<1 mmHg)	1	1–1		1	1–1		1	1–1	
Q2 (1–2.2 mmHg)	1.290	0.752–2.213	0.355	1.11	0.619–1.992	0.726	1.013	0.556–1.847	0.966
Q3 (2.2–4 mmHg)	1.447	0.859–2.435	0.165	1.091	0.618–1.926	0.763	0.775	0.423–1.419	0.409
Q4 (≥4 mmHg)	2.109	1.24–3.587	***0.006***	1.539	0.858–2.763	0.148	1.614	0.89–2.924	0.115
Inter arm DBP> = 5 mmHg	1.917	1.141–3.219	***0.014***	1.464	0.798–2.686	0.218	1.433	0.732–2.808	0.294

Aadjusted for age, sex, BMI, current smoking and drinking status, diabetes, low density lipoprotein, average brachial SBP, heart rate, anti-hypertensive treatment, and statin use. The odds ratio expressed the risk in the ICAS and ECAS group compared with the non-stenosis group. Isolated ECAS, extracranial arterial stenosis only; Isolated ICAS, intracranial arterial stenosis only; COMB, combined extra- and intracranial arterial stenosis.
