# Supplementary material for: Association of Inter-arm Blood Pressure Difference with Asymptomatic Intracranial and Extracranial Arterial Stenosis in Hypertension Patients
Source: Sci Rep. 2016 Jul 14;6:29894. doi: 10.1038/srep29894 (PMC4944122; doi:10.1038/srep29894)

Association of Inter-arm Blood Pressure Difference with Aysmptomatic Intracranial and Extracranial Arterial Stenosis in Hypertension Patients

Yan Wang[1], Jin Zhang[1], Yuesheng Qian[1], Xiaofeng Tang[1], Huawei Ling[2], Kemin Chen[2], Yan Li[1], Pingjin Gao[1], Dingliang Zhu[1*]

1. Research Center for Hypertension Management and Prevention in Community, Shanghai Key Laboratory of Hypertension, Shanghai Institute of Hypertension, State Key Laboratory of Medical Genomics, Ruijin Hospital, Shanghai Jiaotong University School of Medicine, Shanghai, China
2. Department of Radiology, Ruijin Hospital, Shanghai Jiaotong University School of Medicine, Shanghai, China

Tel: +86- 21-64313816

Fax: +86- 21-54654498

Email: Dingliang Zhu: zhudingliang@sibs.ac.cn,

Running title: inter-arm BP difference and ICAS

Supplementary figure 1. Prevalence of severity (%) of ECAS and ICAS stenosis according to inter-arm SBP ≥ 10mmHg (a and b) and DBP ≥ 5mmHg (c and d). ECAS, extracranial arterial stenosis; ICAS, intracranial arterial stenosis.

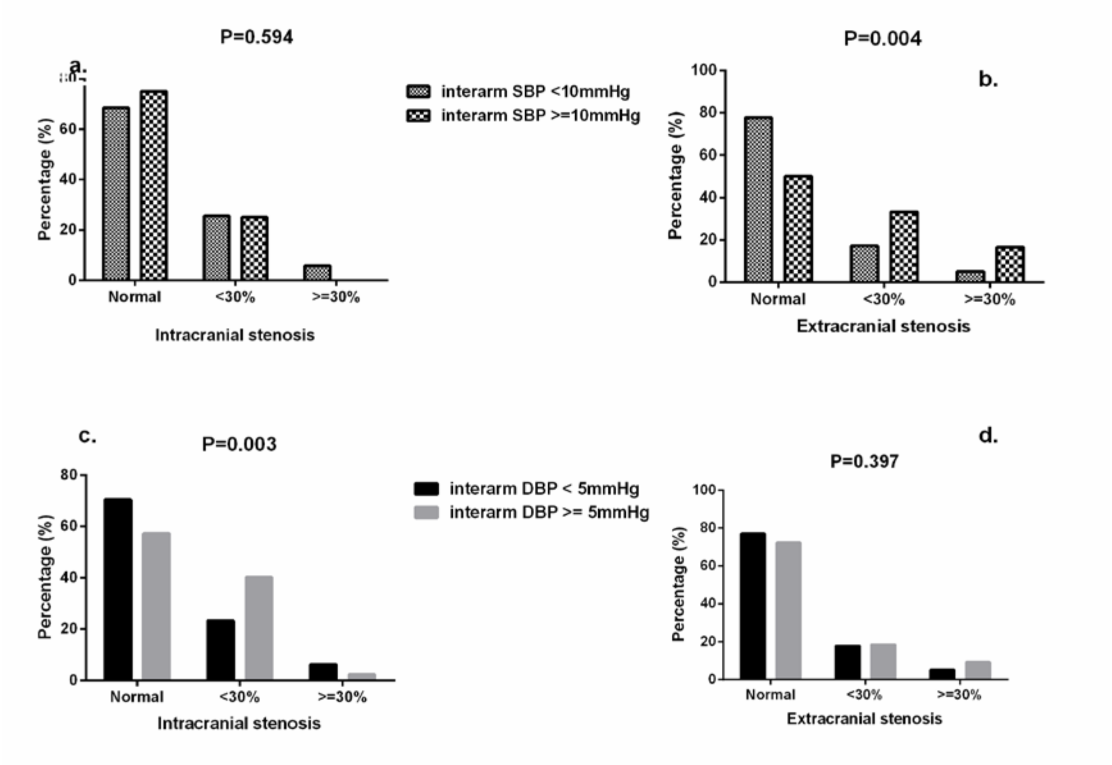

Supplementary figure 2. Prevalence of involved ECAS and ICAS arteries according to inter-arm SBP ≥ 10mmHg (a and b) and DBP ≥ 5mmHg (c and d). ECAS, extracranial arterial stenosis; ICAS, intracranial arterial stenosis.

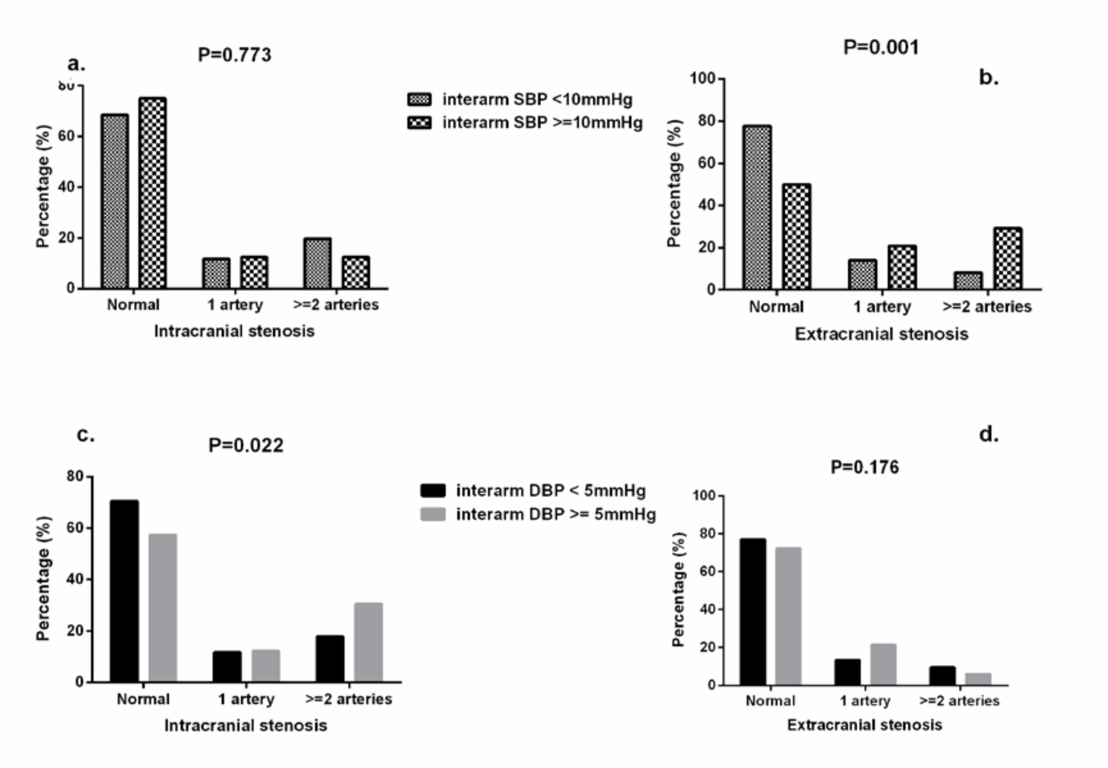

Supplement: Supplementary Information [file srep29894-s1.pdf]
